# *Wolbachia* springs eternal: symbiosis in Collembola is associated with host ecology

**DOI:** 10.1098/rsos.230288

**Published:** 2023-05-31

**Authors:** Jules Rodrigues, Emilie Lefoulon, Laurent Gavotte, Marco Perillat-Sanguinet, Benjamin Makepeace, Coralie Martin, Cyrille A. D'Haese

**Affiliations:** ^1^ UMR7245, MCAM, Museum national d'Histoire naturelle, Paris, France; ^2^ UMR7179 MECADEV, Museum national d'Histoire naturelle, Paris, France; ^3^ School of Animal and Comparative Biomedical Sciences, University of Arizona, Tucson, AZ, USA; ^4^ Espace-Dev, University of Montpellier, Montpellier, France; ^5^ ISEM, University of Montpellier, Montpellier, France; ^6^ Institute of Infection and Global Health, University of Liverpool, Liverpool, UK

**Keywords:** *Wolbachia*, springtails, symbiosis, evolution

## Abstract

*Wolbachia* are endosymbiotic alpha-proteobacteria infecting a wide range of arthropods and nematode hosts with diverse interactions, from reproductive parasites to obligate mutualists. Their taxonomy is defined by lineages called supergroups (labelled by letters of the alphabet), while their evolutionary history is complex, with multiple horizontal transfers and secondary losses. One of the least recently derived, supergroup E, infects springtails (Collembola), widely distributed hexapods, with sexual and/or parthenogenetic populations depending on species. To better characterize the diversity of *Wolbachia* infecting springtails, the presence of *Wolbachia* was screened in 58 species. Eleven (20%) species were found to be positive, with three *Wolbachia* genotypes identified for the first time in supergroup A. The novel genotypes infect springtails ecologically and biologically different from those infected by supergroup E. To root the *Wolbachia* phylogeny, rather than distant other Rickettsiales, supergroup L infecting plant-parasitic nematodes was used here. We hypothesize that the ancestor of *Wolbachia* was consumed by soil-dwelling nematodes, and was transferred horizontally via plants into aphids, which then infected edaphic arthropods (e.g. springtails and oribatid mites) before expanding into most clades of terrestrial arthropods and filarial nematodes.

## 1. Introduction

The alpha-proteobacterium *Wolbachia* (Rickettsiales) is an endosymbiont of two phyla in the Ecdysozoa: arthropods [1] and nematodes (the Onchocercidae family, commonly known as filariae, and the Tylenchida order [2–6]). *Wolbachia* is transmitted vertically from mother to offspring. However, studies on *Wolbachia*–host coevolution suggest that horizontal transfer events from bacteria to a new host have occurred during the evolution of the species [6–8]. Moreover, the lack of congruence between the phylogenies of hosts and those of *Wolbachia* supports the existence of horizontal transmissions on an evolutionary scale [5,6,9,10]. Sequencing of filarial genomes revealed the presence of lateral transfers of *Wolbachia* gene fragments ranging from about 200 bases to more than 3 kb [11–13]. In particular, one study estimated that more than 4.5% of the *Wolbachia* genome from *Brugia malayi* was transferred to the genome of its filarial host [14]. These transfers are not specific to filariae and have also been identified in arthropod genomes [12,15].

One difference between *Wolbachia* symbionts of arthropods and nematodes is the nature of the symbiosis. *Wolbachia* is mainly localized in the reproductive tissues of arthropods and it is responsible for the induction of a number of reproductive alterations including feminization, parthenogenesis, male-killing and cytoplasmic incompatibility (CI). *Wolbachia* induces changes in the reproduction of its host to promote its own transmission [16–18]. However, the phenotypes of *Wolbachia* in arthropods are not limited to parasitism and the bacteria can act as obligate mutualists. In the bedbug *Cimex lectularius*, *Wolbachia* supplements the blood diet by provision of the B vitamins that are deficient, and the nature of the association is described as nutritional mutualism [19,20]. Antibiotic therapies have also revealed a mutualistic relationship between *Wolbachia* and filarial nematodes, as worms lacking their symbionts cease to produce viable embryos and exhibit reduced longevity [21,22]. *Wolbachia* is essential for enabling embryonic development and supporting adult survival, consistent with their location in the female germline and somatic hypodermal cords. However, most of the time the nature of the association is often undefined and in particular it is not established for plant nematodes [23] and poorly understood for springtails, although a link to parthenogentic reproduction has been suggested [24–27].

Based on phylogenetic studies, *Wolbachia* have been divided into distinct lineages known as supergroups [7,28,29]. To date, 19 *Wolbachia* supergroups have been described and labelled from A to U [6,30–32]. Among them, some supergroups have been demonstrated as not valid, such as the supergroup G which is a combination of A and B and supergroup R (symbionts of cave spider species) that was reassigned to A [33,34]. However, the validity of some supergroups is yet to be established (electronic supplementary material, table S1).

Despite the significant number of phylogenetic and taxonomic studies, the evolutionary root of *Wolbachia* is still a subject of scientific debate. One issue is that phylogenetic analysis methods are prone to long-branch attraction artefacts and therefore the origin of infection remains unresolved. The most recent comprehensive phylogeny suggests that L (in plant nematodes), M (aphids) and E (Collembola and mites) are sister clades to all the other *Wolbachia* supergroups [23]. Regarding supergroup E, 29 species of Collembola have been tested for *Wolbachia* up to now and 13 are infected [35–39]. Notably, *Wolbachia* of supergroup E have only been detected in parthenogenetic populations of Collembola [35,36,38,39]. Collembola (springtails) are hexapods (class Entognatha) divided into four orders: Poduromorpha, Entomobryomorpha, Symphypleona, Neelipleona. Their mode of reproduction is either parthenogenetic or sexual. Springtails represent a major group of soil animals, with very high density levels, up to several million individuals per m^2^ in forest soils [40]. Collembola have colonized every environment, climate and latitude (including Antarctica). Their morphology, ecology and biology are diverse and varied. They are characterized by a synapomorphy: a ventral tube on the sternite of the first abdominal segment used for osmoregulation. Their evolutionary history is ancient; they represent some of the first hexapods to appear in the fossil record, at the beginning of the Devonian (400 million years BP) [41]. These small, wingless arthropods have since evolved and diversified into a significant number of species, about 8500 described to date [42] (www.collembola.org). Springtails also participated in the emergence of life from an aquatic environment to a terrestrial environment by colonizing soils [43].

Here, we significantly expand the known diversity of *Wolbachia* in springtails, revealing that different *Wolbachia* supergroups have colonized hosts with divergent ecology and modes of reproduction. Moreover, we infer a comprehensive phylogeny of *Wolbachia* and propose a new hypothesis on the origin of this most prevalent of invertebrate symbionts.

## 2. Material and methods

### 2.1. Specimens and species

The springtails specimens in this study belong to 58 species from France and various locations worldwide (table 1). Each batch was collected from the wild and was recorded with a number in the national collections of the MNHN. No permissions were required prior to conducting this research. Collembola were identified by C. D'Haese using dichotomous reference keys based on morphological characters. Representatives of the four orders of springtails were analysed.

**Table 1.** Springtail species screened for the presence of *Wolbachia* in this study. Fifty-eight Collembola species are present. One to three specimens per species were individually screened for *Wolbachia.* The seventh column indicates the Muséum National d'Histoire Naturelle Paris registration number. The eighth column indicates the springtail reproduction mode: ‘parthenogenetic’, ‘bisexual’ (sexual or parthenogenetic depending on populations) or unknown ‘?’. The last column shows the *Wolbachia* infected springtail species (+). Wb, *Wolbachia.*

order	family	genus	species	country origin	sample localization	id sample	reproduction	Wb ?
Entomobryomorpha	Coenaletidae	*Coenaletes*	*carribaeus*	France	Martinique	ma103-w45	bisexual	−
Entomobryomorpha	Entomobryidae	*Entomobrya*	*multifasciata*	France	Jardin des plantes, MNHN	c151113d-w7	bisexual	−
Entomobryomorpha	Entomobryidae	*Entomobrya*	*nivalis*	Italy	Toscana (Siena)	ita01-w62	?	−
Entomobryomorpha	Entomobryidae	*Heteromurus*	*major*	France	Jardin des plantes, MNHN	c151113d-w6	bisexual	−
Entomobryomorpha	Entomobryidae	*Heteromurus*	*nitidus*	Norway	Manche	c151223c-w21	bisexual	−
Entomobryomorpha	Entomobryidae	*Lepidocyrtus*	*lanuginosus*	France	Fontainebleau Forest	c160612-w26	bisexual	−
Entomobryomorpha	Entomobryidae	*Lepidocyrtus*	*lignorum*	France	Svalbard	sva19-w67	bisexual?	−
Entomobryomorpha	Entomobryidae	*Lepidocyrtus*	sp.	France	Manche	c160821a-w34	bisexual	−
Entomobryomorpha	Entomobryidae	*Orchesella*	*cincta*	France	Manche	c151113d-w8	bisexual	−
Entomobryomorpha	Isotomidae	*Agrenia*	*bidenticulata*	Norway	Svalbard	sva53b-w60	bisexual	−
Entomobryomorpha	Isotomidae	*Cryptopygus*	*sverdrupi*	Antarctic	Dronning Maud Land	ata057-w59	bisexual	−
Entomobryomorpha	Isotomidae	*Folsomia*	sp.	France	Manche	c160821a-w38	?	+
Entomobryomorpha	Isotomidae	*Hemisotoma*	*cf thermophila*	France	Manche	c160821a-w41	?	+
Entomobryomorpha	Isotomidae	*Isotoma*	*viridis*	France	Manche	c151223b-w18	bisexual	−
Entomobryomorpha	Isotomidae	*Parisotoma*	*notabilis*	France	Brunoy	sasa002-w16	parthenogenetic	+
Entomobryomorpha	Tomoceridae	*Pogonognathellus*	*flavescens*	Italy	Emilia-Romagna (Piacenza)	it05-w63	bisexual	−
Entomobryomorpha	Tomoceridae	*Tomocerus*	*minor*	France	Manche	c160821a-w33	bisexual	−
Neelipleona	Neelidae	*Megalothorax*	*minimus*	France	Manche	c151223b-w20	parthenogenetic	+
Neelipleona	Neelidae	*Megalothorax*	*laevis*	French Guyana	Nouragues	guy004b-w55	parthenogenetic	+
Neelipleona	Neelidae	*Megalothorax*	*willemi*	France	Jardin des plantes, MNHN	c151113a-w17	parthenogenetic	−
Neelipleona	Neelidae	*Neelus*	*koseli*	Slovakia		svk091120-w57	parthenogenetic	+
Poduromorpha	Brachystomellidae	*Brachystomellides*	*navarinensis*	Chile	Patagonie	c273a1-w48	bisexual	−
Poduromorpha	Brachystomellidae	*Brachystomellides*	*neuquensis*	Chile	Patagonie	chl230-w71	bisexual	−
Poduromorpha	Hypogastruridae	*Ceratophysella*	*denticulata*	New Zealand	Forêt de Taruara	nz239ms-w51	bisexual	−
Poduromorpha	Hypogastruridae	*Hypogastrura*	*cf. subboldorii*	France	Manche	c091225-w10	?	−
Poduromorpha	Hypogastruridae	*Hypogastrura*	*tullbergi*	Norway	Svalbard	sva53b-w61	?	−
Poduromorpha	Hypogastruridae	*Microgastrura*	*massoudi*	France	Nouvelle-Calédonie	ncl057-w49	bisexual?	−
Poduromorpha	Hypogastruridae	*Triacanthella*	*clavata*	Chile	Parque Nacional Torres del Paine	chlc259a-w47	bisexual	−
Poduromorpha	Hypogastruridae	*Triacanthella*	*madiba*	South Africa	Table Mountain	zaf125-w56	bisexual	−
Poduromorpha	Hypogastruridae	*Triacanthella*	sp.	Australia	Lord Howe Island	aus021cj-w42	bisexual	−
Poduromorpha	Hypogastruridae	*Xenylla*	*grisea*	France	Fontainebleau Forest	c151223c-w23	bisexual	−
Poduromorpha	Hypogastruridae	*Xenylla*	sp.	France	Manche	c160821a-w35	?	−
Poduromorpha	Hypogastruridae	*Xenylla*	*szeptyckii*	France	Jardin des plantes, MNHN	c120606-w12	bisexual	−
Poduromorpha	Neanuridae	*Anurida*	*granaria*	New Zealand	Kaimai Mamaku Forest	nzl111-w58	?	−
Poduromorpha	Neanuridae	*Anurida*	*maritima*	Netherlands	Texel	nldn207-w14	bisexual	+
Poduromorpha	Neanuridae	*Bilobella*	*braunerae*	France	Manche	c160821a-w37	bisexual	−
Poduromorpha	Neanuridae	*Caledonimeria*	*mirabilis*	New Caledonia	Koghis	nc032-4-w79	bisexual?	−
Poduromorpha	Neanuridae	*Delamarellina*	*cf ubiquata*	New Zealand	Turuara Forest	nzltararua101124-w69	?	−
Poduromorpha	Neanuridae	*Friesea*	*najtae*	Australia	southwest	aus021a-w44	bisexual	−
Poduromorpha	Neanuridae	*Holacanthella*	*brevispinosa*	France	Jardin Entomologie, MNHN	nzlwhakapapams-w46	bisexual	−
Poduromorpha	Neanuridae	*Monobella*	*grassei*	France	Jardin des plantes, MNHN	c151113d-w4	bisexual	−
Poduromorpha	Neanuridae	*Neanura*	*muscorum*	France	Jardin des plantes, MNHN	c170321-w75	parthenogenetic	−
Poduromorpha	Neanuridae	*Neotropiella*	*carli*	French Guyana	Nouragues	guy006-w52	bisexual	−
Poduromorpha	Neanuridae	*Pronura*	*gaucheri*	French Guyana	Nouragues	guy038-w54	bisexual	−
Poduromorpha	Neanuridae	*Thaliabella*	sp.	Nouvelle-Calédonie	Mont Panié	trb058montpanie-w50	?	−
Poduromorpha	Onychiuridae	*Megaphorura*	*arctica*	Norway	Svalbard	sva20-w66	bisexual	−
Poduromorpha	Onychiuridae	*Oligaphorura*	*groenlandica*	Norway	Svalbard	sva26-w68	parthenogenetic	+
Poduromorpha	Onychiuridae	*Protaphorura*	sp.	France	Manche	cdh308-w70	bisexual	−
Poduromorpha	Pachytullbergiidae	*Pachytullbergia*	*scabra*	Argentina	Quetrihué peninsula	arg140-w53	bisexual	−
Poduromorpha	Poduridae	*Podura*	*aquatica*	Netherlands	Texel	nldn211-w13	bisexual	+
Poduromorpha	Tullbergiidae	*Mesaphorura*	sp.	France	Fontainebleau Forest	sasa001-w15	parthenogenetic	+
Symphypleona	Dicyrtomidae	*Dicyrtoma*	*fusca*	France	Manche	c151222b-w24	bisexual	−
Symphypleona	Dicyrtomidae	*Dicyrtomina*	*saundersi*	France	Manche	c181101b-w ?	bisexual	−
Symphypleona	Katiannidae	*Sminthurinus*	*aureus*	France	Manche	cdh304-w65	bisexual	−
Symphypleona	Katiannidae	*Sminthurinus*	*cf niger*	France	Manche	c090923-w64	bisexual	−
Symphypleona	Sminthurididae	*Sminthurides*	*cf aquaticus*	France	Manche	c151223b-w19	bisexual	−
Symphypleona	Sminthurididae	*Sminthurides*	*cf concolor*	France	Manche	c160821b-w31	bisexual	−
Symphypleona	Sminthurididae	*Sphaeridia*	*pumilis*	France	Manche	C181101a-w31	bisexual	+

### 2.2. Molecular screening

The DNA extraction was performed on individual specimens. One to three specimens per species were analysed, representing 58 Collembola species (table 1). The Qiagen (Hilden, Germany) DNeasy tissue extraction kit was used for the extractions. Tissues were digested in lysis buffer and proteinase K at 56°C for 3 h, then extraction was carried out as advised by the manufacturer. Total DNA was finally resuspended in 100 µl of the elution buffer.

The presence of *Wolbachia* was screened on the 95 specimens. *Wolbachia* symbionts were characterized by nested PCR screening of the six genetic markers (16S rDNA, *ftsZ*, *dnaA*, *coxA*, *fbpA* and *gatB*) (electronic supplementary material, table S2). PCR products were purified and sequenced by Eurofins Genomics using the Sanger method. Chromatograms were analysed and edited using CodonCode Aligner. Supergroups of *Wolbachia* were identified as described in previous studies [5,6,29]. A total of 45 sequences were deposited in the GenBank Data Library: OQ857548 to OQ857552 for 16S ADNr sequences and OQ859108 to OQ859146 for the other genetic markers (electronic supplementary material, table S3).

### 2.3. Phylogenetic reconstruction

To analyse the newly obtained sequences, a dataset of GenBank sequences was built. *Wolbachia* sequences from all valid supergroups were extracted from the GenBank database for the six genetic markers (16S rDNA, *ftsZ*, *dnaA*, *coxA*, *fbpA* and *gatB*). These sequences were then reviewed to delete contaminated or misassigned sequences. Taxa with only one genetic marker available were not included in the dataset. The dataset of GenBank sequences consisteds of 100 taxa (electronic supplementary material, table S3). The selected outgroups are other Rickettsiales belonging to the genera *Anaplasma* spp. *(A. centrale* and *A. marginale*) and *Erlichia* spp. (*E. chaffeensis* and *E. ruminantium*) or the *Wolbachia* genotypes of the supergroup L infecting *Pratylenchus penetrans* and *Radopholus similis*.

Sequences were aligned, for each locus independently, using MAFFT v7.505 [44] with default parameters. Alignment of coding sequences was optimized to consider all three codon frames. PhyloSuite [45] was used to make the final concatenated alignment. It comprises 4751 bp for 114 terminals and was analysed using maximum likelihood (ML), with 1000 bootstraps, implemented in the program RAxML v. 8.2.12 [46] under GTRCAT model. The concatenated dataset was also analysed using Bayesian inference (BI) using BEAST v. 2.6.6 [47] with best fit nucleotide substitution models for each partition determined using bModelTest and all partitions estimated with the lognormal relaxed clock (uncorrelated) and Yule process tree prior.

## 3. Results and discussion

### 3.1. *Wolbachia* and springtails

Out of the 58 studied species of springtails, the presence of *Wolbachia* was detected with the amplification of at least one genetic marker in 11 species. Among them, six are new genotypes of *Wolbachia* (table 1). Thus, about 20% of the collembolan species in this study were found to be infected by the bacteria*.* This prevalence is lower than those described in previous studies with 4/6 (66%) [36]; 3/9 (33%) [38]; and 4/11 (36%) [39]. The difference in prevalence may be explained by the sample size, which in this study is five times higher than the previous largest one (11 springtails species [39]). Hence, the dataset of the current study should be less prone to sample bias.

**Figure 1.**
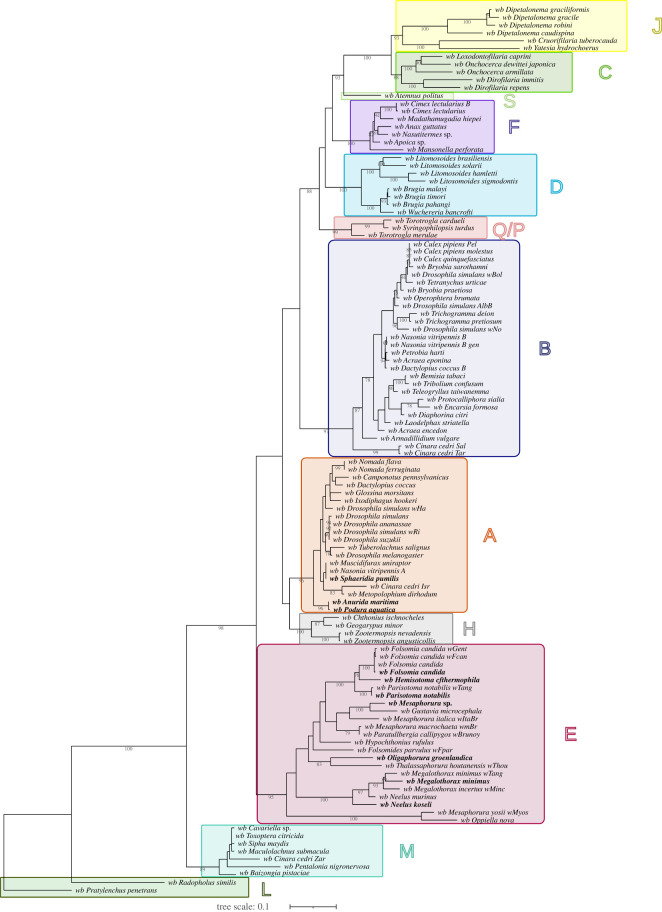
Phylogenetic tree of *Wolbachia* on six markers by ML with supergroup L used to root the tree. Analysis based on partitioned concatenation of 16S rDNA, *dnaA*, *ftsZ*, *coxA*, *fbpA* and *gatB* sequences. The total length of datasets is approximately 4750 bp. 114 *Wolbachia* genotypes were analysed. The topology was inferred using ML inference using RaxML v. 8.2.12. Nodes are associated with bootstrap values based on 1000 replicates. Bootstraps with values inferior to 75 are not displayed. The scale bar indicates the distance in substitutions per nucleotide. Each colour is associated with a supergroup: dark green, L; teal blue, M; red, E; grey, H; orange, A; deep blue, B; light blue, D; purple, F; light green, S; yellow, J; green, C. wb, *Wolbachia*.

**Figure 2.**
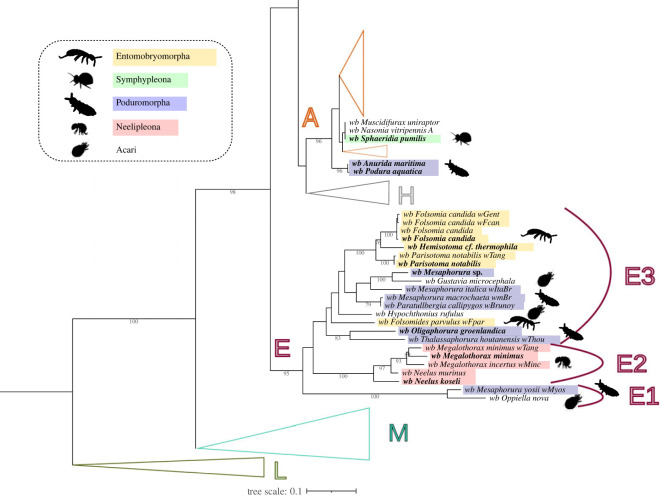
Focus on the *Wolbachia* phylogenetic clades harboured by springtails. Figure 1 is used to emphasize the *Wolbachia* infecting springtails thus focusing on supergroups A and E. Colours and animal drawings represent the springtail orders (green: Symphypleona; red: Neelipleona; yellow: Entomobryomorpha; blue: Poduromorpha). The *Wolbachia* genotypes in bold have been screened in the current study. The supergroup E is divided into three clades, E1, E2, and E3, to facilitate the discussion.

The genotypes of *Wolbachia* infecting Collembola are distributed in two separate clades: supergroup A and supergroup E (figures 1 and 2). The *Wolbachia* of supergroup E infecting the springtails are more diverse than previously described [36,38,39]. With the addition of these 11 new genotypes, supergroup E is now subdivided into three clades: E1, E2 and E3 (figure 2). The E1 clade consists of *Wolbachia* genotypes infecting the springtail *Mesaphorura yosii* (*w*Myos) and the mite *Opiella nova* (*w*Onov)*.* Previously, *w*Onov was not associated with a supergroup, and it was positioned at the root of the filarial and arthropod supergroup dichotomy [48]. Regarding *w*Myos*,* it was already inferred as the sister group of the remainder of supergroup E [39]. However, *w*Onov and *w*Myos do not have every genetic marker available, sharing only the 16S rDNA and *fbpA* genes. Thus, the E1 clade lacks robustness. The *Wolbachia* genotypes of E2 form a strongly supported clade. Interestingly, the springtails of Neelipleona order are strictly infected by the E2 genotypes, although only two genera are represented in this study (*Megalothorax* spp. and *Neelus* spp.). With two springtail orders and two from Acari infected, the E3 clade is the most diverse of the three E subclades.

**Figure 3.**
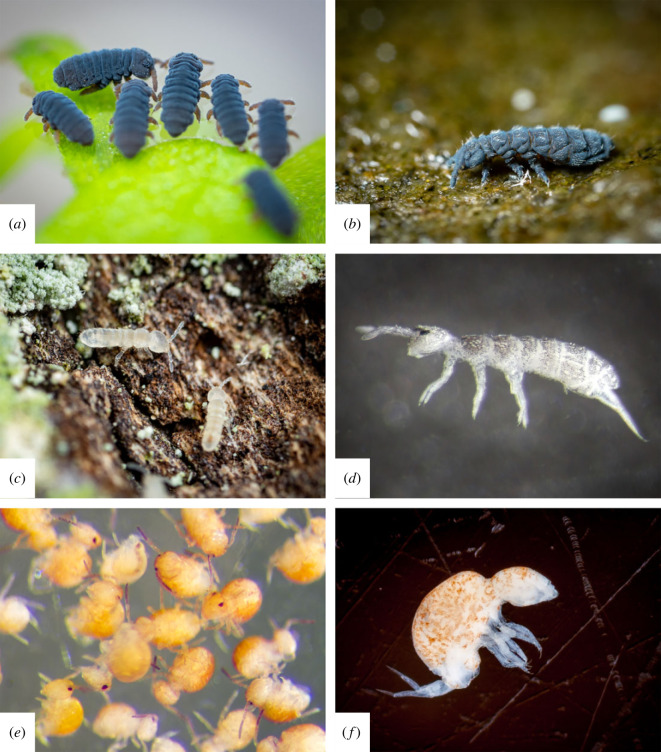
Images of *Wolbachia*-infected springtails illustrating their diversity of pigmentation and forms. (*a*) *Podura aquatica* Linnaeus, 1758 (Poduridae, L.: 1 mm) (sexual reproduction and semi-aquatic). (*b*) *Anurida maritima* (Guérin, 1938) (Neanuridae, L.: 3 mm) (sexual reproduction and semi-aquatic). (*c*) *Folsomia candida* Willem, 1902 (Isotomidae, L.: 1.2 mm) (bisexual reproduction and edaphic). (*d*) *Parisotoma notabilis* (Schäffer, 1896) (Isotomidae, L.: 0.8 mm) (bisexual reproduction and edaphic). (*e*) *Sphaeridia pumilis* (Krausbauer, 1898) (Sminthurididae, L.: 0.5 mm) (sexual reproduction and atmobiotic). (*f*) *Neelus koseli* Kovác & Papác 2010 (Neelidae, L.: 0.6 mm) (bisexual reproduction and edaphic).

The springtails infected by the genotypes of the supergroup E are euedaphic: they live in the soil, are small and have lost their pigmentation (figure 3*c–e*) [40,49]. Moreover, their mode of reproduction is either parthenogenetic or unknown (table 1). Parthenogenesis, by making obsolete the need to find a mate, facilitates the exploration of new underground environments, thus extending the distribution area of the population [50]. One of the *Wolbachia* of supergroup E, the genotype infecting *Folsomia candida* (*w*Fol), has unique genomic characteristics compared to other genomes of *Wolbachia*. While the depletion of *Wolbachia* in haplo-diploid arthropod hosts leads to the development of males, in *F. candida* the presence of antibiotics strongly limits the production of eggs and once the treatment ends, the egg production recovers to its normal level, suggesting *w*Fol is able to enter a reversible persister state [25–27,51]. Bacterial toxin–antitoxin (TA) modules are present in all of the genomes of *Wolbachia* studied infecting arthropods [52]. The expression of TA modules leads to the synthesis of either a toxin interfering with the bacterial cell growth or an antitoxin to neutralize the associated toxin. These modules are involved in three functions: post-segregational killing, abortive infection and protection against environmental stress, such as antibiotics [53]. These TA modules would be one of the genetic pathways leading to persister state with the activation of TA toxins inhibiting vital processes. One of the TA modules present in the *w*Fol genome encodes for an Abi (abortive infection) Type IV TA system and has only been found in this genome [52]. However, the function of this module in *Wolbachia* has yet to be deciphered. One hypothesis would be the involvement in the reversible persister state of *w*Fol.

Three *Wolbachia* infecting springtails were classified in supergroup A. Among them, the presence of a genotype infecting *Sphaeridia pumilis* is also the first observed case of a symphypleonan infected by *Wolbachia*, indicating that all four springtail orders (Entomobryomorpha, Poduromorpha, Neelipleona and Symphypleona) are infected by this bacterium. All the springtail species infected by *Wolbachia* from supergroup A live at the surface either in semi-aquatic environments (*Anurida maritima* and *Podura aquatica*) [54,55] or in moist environments (*S. pumilis*) [56]. They have a high metabolism and are highly pigmented (figure 3) [57]. More importantly, these three Collembola species have a sexual mode of reproduction (table 1).

In springtails, infections either by *Wolbachia* of supergroup E or supergroup A seem strongly correlated with their ecology.

### 3.2. The use of an outgroup with *Wolbachia*: no perfect solution in sight

In this study, two different outgroups have been assigned to infer the *Wolbachia* phylogeny. First, the usual Rickettsiales outgroups (electronic supplementary material, table S4), *Ehrlichia* and *Anaplasma*, were used (electronic supplementary material, figure S1). Second, the phylogeny was inferred with the supergroup L as the outgroup (figure 1; electronic supplementary material, figure S2).

Usually, an outgroup should be selected from outside the studied phylum, but the other Rickettsiales are too phylogenetically distant. Inferring *Wolbachia* phylogenies with *Anaplasma* and *Ehrlichia* might generate long-branch attraction artefacts and multiple polytomies [3,5,8,10,23,32,33,35,36,48,58–74]. This is why more than half of the produced topologies of *Wolbachia* do not use an outgroup (electronic supplementary material, table S4). However, when the topology is rooted and supergroup L is present in the phylogeny, in 14 out of 18 phylogenies (82%), supergroup L is consistently positioned as the sister group of the remaining *Wolbachia* (electronic supplementary material, table S4).

Supergroup L *Wolbachia* infects plant-parasitic nematodes (PPN) and up to now three genotypes have been described. The first two genotypes are known to infect nematode hosts: *Radopholus similis* (*w*Rad) [63] and *Pratylenchus penetrans* (*w*Ppe) [3]. The third one is an assembly of six *Wolbachia*-positive samples originating from the same soil of a Texan farm (USA). The contigs obtained from the sequencing of the six *Wolbachia*-positive samples were highly similar between themselves. Thus, to increase the coverage of the assembly, the authors decided to pool the 192 contigs and named the final assembly *w*Tex [23]. Regarding the hosts of *w*Tex, two genera might be associated this pool: *Helicotylenchus* spp. and *Rotylenchus* spp. The genomes of *w*Rad, *w*Ppe and *w*Tex share multiple characteristics [3,23], most notably a lack of *cifA* and *cifB* genes (which are linked to cytoplasmic incompatibility i), WO phage, and homologues of biotin synthesis genes. These absent features make these genotypes closer to the *Wolbachia* infecting filariae (supergroups C, D, J) than those infecting arthropods (e.g. supergroups A and B). However, the core genes of *w*Rad, *w*Ppe and *w*Tex share the most similarities with the core genes of other *Wolbachia* infecting plant-feeding arthropods such as *Bemisia* spp., *Bryobia* spp*.* or *Cinara* spp*.* [3,23] The shared genetic (the conserved glycolysis and nucleotides biosynthesis pathways) and genomic (%GC, orthologue length, ankyrin repeat proteins) features support the inclusion of supergroup L in the *Wolbachia* lineage [3,23]. Importantly, whereas *Anaplasma* spp. and *Ehrlichia* spp. are bacteria infecting hematophagous ticks, the most anciently derived *Wolbachia* supergroups (L, M, E, H) infect hosts dwelling in the soil and/or arthropod plant pests, which are ecologically closer to PPN. The supergroup organization in the phylogeny with supergroup L as outgroup (figure 1; electronic supplementary material, figure S2) are highly similar to the one with Rickettsiales as outgroup (electronic supplementary material, figure S1). Briefly, supergroup M is the closest to supergroup L, and supergroup E is positioned between supergroup M and the rest of the *Wolbachia* supergroups (figure 1; electronic supplementary material, figure S1). Supergroups A and H form a clade in figure 1, while in these supergroups are paraphyletic (electronic supplementary material, figure S1). Another difference between these two phylogenies is the positioning of supergroup Q/P: while rooted with supergroup L, it is a sister clade of filarial supergroups (D, F, C, J) and supergroup S but when rooted with *Erlichia* spp. and *Anaplasma* spp. it forms a clade with supergroup F. However, the main difference between these two phylogenies is the distribution of the cladogenesis events. In the phylogeny with the Rickettsiales (electronic supplementary material, figure S1), the cladogenesis events are distally aggregated while in the phylogeny rooted in L, the cladogenesis events are more evenly distributed (figure 1). The latter enables a deeper resolution of the evolutionary distance between the supergroups. Therefore, using supergroup L to root and polarize the *Wolbachia* phylogenies is justified, even though it does not follow the conventional definition of an outgroup.

### 3.3. An evolutionary hypothesis: from soil to the tree?

**Figure 4.**
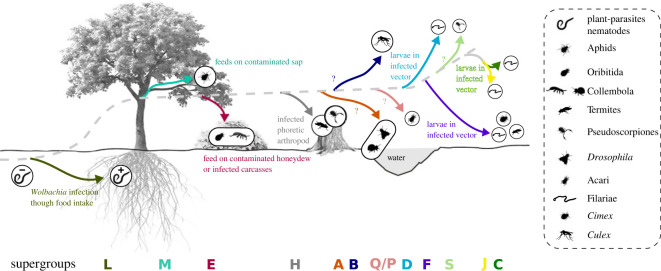
An illustrated hypothesis for *Wolbachia* evolution based on the integration of ecological and biological factors. The dashed grey line represents the evolution of *Wolbachia*, polarized from the least (left) to the most (right) recently derived clades. Each coloured arrow represents a hypothetic host-switching event toward an actual supergroup. The colour code is the same as figure 1. The hosts are represented in their ecological niche: *Wolbachia*-free nematoda are in the soil; plant–parasite nematoda infected by supergroup L are in roots; aphids infected by supergroup M are on leaves; springtails and Oribitida mites infected by supergroup E are in soil litter; termites and pseudoscorpiones infected by supergroup H are respectively in dead wood and in a phoretic interaction; springtails infected by supergroup A are close to water. Suggested ways of *Wolbachia* infection are written along arrows.

The evolutionary history of *Wolbachia* is influenced by two opposite processes: (i) bacteria coevolve with their host or shift to new hosts and (ii) the host becomes extinct, or symbiont loss occurs [75]. The latter process hinders the possibility of determining a comprehensive evolutionary history of *Wolbachia*, with host shift events between supergroups whose existence has in most cases been lost. Though ‘paleosymbiosis’ may be inferred with the detection of nuclear *Wolbachia* transfers in *Wolbachia*-free host genome [76]. With this caveat in mind, it is still possible to try to decipher how *Wolbachia* shifted hosts in order to attain the current supergroup distribution (figure 4). Sanaei *et al.* [75] hypothesized that four steps are required for a successful host shift of *Wolbachia*: (i) a physical transfer of the endosymbiont to a new species must occur, through a predator–prey interaction, or a host–parasite interaction, or by sharing plant and other food sources; (ii) the bacteria must be able to develop inside the new host; (iii) a maternal transmission of *Wolbachia* must be possible; and (iv) *Wolbachia* must successfully spread in the new host population.

Up to now, supergroup L is the sister group of all the other supergoups (figure 1). It is commonly accepted that Nematoda lost their ability to synthesize heme and they acquire it from their bacterial diet [77,78]. However, the root system is deprived of heme and *Wolbachia* has an intact heme synthesis pathway. Thus, Weyandt & Aghdam [23] hypothesized that the ancestral *Wolbachia–*PPN relationship enabled the PPN ancestor to shift from bacterivory to plant parasitism (figure 4). Genes currently linked with the manipulation of the reproduction phenotype have not been yet detected in these *Wolbachia*. Thus, the ancestral state of the *Wolbachia* symbiosis might not be a reproductive parasite but rather a nutritional symbiont. However, given the diversity of *Wolbachia* relationships, more studies are needed to characterize the *Wolbachia*–PPN symbiosis.

Close to supergroup L is supergroup M, which until now has only been detected in sap-sucking aphids. Cases of horizontal transfer to an aleyrodid host, the whitefly *Bemisia tabaci*, via the plant environment have been documented [79]: *Wolbachia*-positive specimens of *B. tabaci* were put in contact with cotton plants for their diet. After 15 days the sap-sucking insects were removed. Then, a *Wolbachia*-specific FISH visualization was used to observe the presence of the bacteria in the leaves. Interestingly, the bacteria were present not only in the infested leaves, but also in the phloem and in leaves which were not in direct contact with the whiteflies. *Wolbachia* was still present in the plant for at least 50 days after the removal of the *Wolbachia*-positive whiteflies. Moreover, when *Wolbachia*-free whiteflies fed on leaves contaminated with *Wolbachia*, they in turn became infected. Thus, *Wolbachia* is still active after residing in the plant environment, making a horizontal transfer of *Wolbachia* from PPN to aphids via the plant a plausible event (figure 4). Contrary to the PPN, aphids are in an ancient mutualistic association with another bacteria: *Buchnera* (around 200 million years (Ma) old [80]). The *Buchnera*–aphid coevolution is sufficiently ancient for the *Buchnera* genome to have become highly reduced (from 600 kb to 400 kb) and to have lost key metabolic functions, such as the biosynthesis of peptidoglycan or nucleotides [81]. If it has been largely demonstrated that *Buchnera* presents a mutualistic association with their aphid host by providing essential amino acids, the role of *Wolbachia* in aphid remains a matter of discussion [82,83]. A study has suggested that the apparent fixation of *Wolbachia* in populations of aphids *Pentalonia nigronervosa* might be because *Wolbachia* and *Buchnera* symbionts complement each other in several important pathways to provide essential amino acids and vitamins to the aphid host [83]. However, a new analysis of the genomic data and disagreement on the interpretation of the antibiotic treatment experiment highlighted that there is not enough evidence of *Wolbachia* being a nutritional co-obligate in this aphid [82]. Thus, the role of Wolbachia of the supergroup M in the association with aphids is yet to be deciphered.

Honeydew is excreted by aphids and can fall on soil, promoting the growth of microbial biomass and attracting fungivorous and bacterivorous springtails [84]. The oribatid mites (Acari) infected by supergroups E share the same habitat and have a similar food source to these springtails [67]. Thus, the microorganism hotspot induced by the presence of honeydew may have favoured horizontal transfer between springtails and mites of these supergroups (figure 4). In aphids, *Wolbachia* can be either scattered in different tissue or confined in bacteriomes in the abdomen [85,86]. Given the presence of bacteria in honeydew [87], the horizontal transfer from M to E may have occurred through this way. Another hypothesis to explain the hypothetically horizontal transfer between supergroups M and E would be linked to the detritivore activity of the springtails. In 2019 Feng *et al.* [88] marked insect carcasses and fungi decomposing these carcasses with stable isotopes to observe the diet preference of the Collembola. In their study, the springtails favoured the consumption of insect carcasses, indicating springtails may ingest arthropod carcasses in the wild. Moreover, the study of Brown & Lloyd [89], where *Wolbachia*-free mites became infested by the bacteria by consuming fly corpses, indicates arthropods may become infected by *Wolbachia* via detritivory.

The evolutionary history of the supergroups positioned as sister clades of the supergroup E is complex. The origin of infection of supergroup H (figure 4), composed of *Wolbachia* infecting termites and pseudoscorpions, may be explained by an ancient springtail relationship with social insects. In an approximately 16 Ma old amber fossil, springtails were observed grasping a winged termite and an ant, apparently in a phoretic association [90]. This close contact may have been the vector of the ancient horizontal transfer of *Wolbachia* between springtails and termites. Moreover, pseudoscorpions are also phoretic with other insects [91], so there might have been a horizontal transfer of *Wolbachia* when springtails and pseudoscorpions were in phoresy with the same termite host. In supergroups A and B numerous horizontal transfers [1,92,93] and secondary losses [94] occurred. Various phenomena may be involved to explain these host-switches [75]: predator–prey interactions, host–parasitoid interactions, spatial proximity, host hybridization with another species and shared trophic interactions. The presence of *Wolbachia* infecting sexual springtails in supergroup A underlines the complexity of the evolutionary history of these bacteria. Their origin of infection may be linked to their ecological niche, but the nature of their interaction has yet to be deciphered.

### 3.4. Optimizing the use of genetic markers

Published topologies including the supergroups E, M or L were analysed to better understand how to calibrate the *Wolbachia* phylogeny. Close to half of the cladograms were inferred with one gene (table 2), mostly with the 16S rRNA gene (20 out of 31), which has the advantage of being the most represented *Wolbachia* gene in the databases (electronic supplementary material, table S4). Therefore, the record of the highest number of genotypes (*n* = 236) is held by a phylogeny inferred with this single gene [65]. However, the topologies inferred with solely this gene often have multiple polytomies and supergroups incoherently positioned [8,65,71,74,95,96].

**Table 2.** Classification of *Wolbachia* topologies according to the number of studied genes containing at least the supergroups E, M, or L. The topologies are distributed into five categories depending on the number of genetic markers used (column 1, ‘number of genes’). For each category the number associated is indicated (column 2, ‘occurrences’). Column 3 indicates the number of genotypes (median with minimum and maximum values). The last column indicates the number of supergroups (median with minimum and maximum values).

number of genes	occurrences	number of genotypes	number of supergroups
1	31	30 (14–236)	7 (5–16)
2	7	55 (36–68)	8 (7–10)
3	8	31.5 (12–59)	7 (6–15)
>3	11	33 (21–145)	9 (7–17)
proteins/genome-wide	15	21 (16–90)	7 (6–12)

Although 16S rDNA is the most sequenced gene, the phylogenies with the highest number of taxa were inferred with two genes (table 2). This discrepancy may be explained by the year of publication of these topologies, with half of the one-gene topologies published before 2009, while half of the topologies with two genes were published after 2015 (electronic supplementary material, table S4). Before 2009, fewer genotypes were available, with the largest phylogeny having 59 taxa [36], whereas in 2015 the largest phylogeny had 236 genotypes, but 109 of these genotypes are *Wolbachia* of the supergroups A, B and M infecting Chinese aphids [65]. Thus, this phylogeny has a consequent sample bias. This would mean that, on average, the datasets for the phylogenies with two genes had more genotypes available than the phylogenies with only one gene.

To ensure a reliable and robust estimation of the relationships and especially the relevance of the clades/supergroups, the concatenation of multiple genetic markers with various evolutionary rates is needed [97,98]. The genetic markers used for the MLST (multi locus sequence typing) approach (*gatB, coxA, hcpA, ftsZ* and *fbpA*) [7] are widely used to infer multi-gene phylogenies (table 2; electronic supplementary material, table S4). Additionally, 75% of the phylogenies also include the 16S rDNA gene marker (electronic supplementary material, table S4), which has a slower evolutive rate than the MLST genes. However, these genes poorly resolve genotypes, which is particularly detrimental for discrimination within supergroups A and B, in which infections are more recent [98]. Indeed, the genetic markers were selected to allow a classification by the similarity of their allelic profile [7], not for concatenation of markers covering the evolutionary rates of a spectrum of the different phylogenetic levels of *Wolbachia,* and they were designed solely on *Wolbachia* genotypes infecting arthropods. Thus, different authors calculated the recombination rates of the MLST genes to check whether their use was pertinent to discriminate the different supergroups of *Wolbachia* [64,97,98], and the consensus was this was the case.

If one objective of a study is to explore the intra-supergroup relationships where recent infection occurred, such as in supergroups A, B or F, then complementing the MLST genes with other orthologous genetic markers having a higher evolutionary rate would be needed [98]. This has led to increasing interest in whole-genome approaches to generate a sample set of genetic markers with a higher diversity of evolutionary rates. However, the use of *Wolbachia* phylogenomics involves numerous drawbacks: (i) the poor terminal sampling with the lack of data for some supergroups (H, K, N, O, P, Q; electronic supplementary material, table S4) and (ii) lower supergroup intra-diversity than in the phylogenies with multiple genes (electronic supplementary material, table S4). These problems are linked to the cost and the technical difficulties of sequencing *Wolbachia*'s genome. Indeed, currently, the bacteria cannot be cultivated in an axenic culture system [99], thus making a metagenomic approach obligatory. Although *Wolbachia* has a small genome, from 550 kb [4] to 2.19 Mb [100], the presence of many transposal elements, prophage genes and repeat domains makes the assemblies potentially more fragmented [101]. These complex genomic regions are difficult to resolve for short-read sequencing; however, these hurdles may be overcome using a long-read sequencing approach.

## 4. Conclusion

Altogether the evolutionary history of *Wolbachia* genotypes infecting springtails is not straightforward. The nature of the infection depends on the host's biology and ecology: the infection of parthenogenetic soil-dwelling springtails is high and belongs to supergroup E; sexually reproducing springtails living in wet environment are infected by another group of *Wolbachia* (A). The *Wolbachia* of supergroup E are known to be involved in the manipulation of their host reproduction. The nature of the interaction between *Wolbachia* of supergroup A and their springtails hosts will have to be deciphered. Increasing the number of genotypes in sexual springtails would give a better picture of the infection in these arthropods.

## Data Availability

The GenBank accession numbers of the sequences used and produced in this study are available in the electronic supplementary material, table S3 [102].
